# Multilevel network meta‐regression for population‐adjusted treatment comparisons

**DOI:** 10.1111/rssa.12579

**Published:** 2020-06-07

**Authors:** David M. Phillippo, Sofia Dias, A. E. Ades, Mark Belger, Alan Brnabic, Alexander Schacht, Daniel Saure, Zbigniew Kadziola, Nicky J. Welton

**Affiliations:** ^1^ University of Bristol UK; ^2^ University of York and University of Bristol UK; ^3^ Eli Lilly and Company Windelsham UK; ^4^ Eli Lilly and Company Sydney Australia; ^5^ Eli Lilly and Company Bad Homburg Germany; ^6^ Eli Lilly and Company Vienna Austria

**Keywords:** Effect modification, Indirect comparison, Individual patient data, Network meta‐analysis

## Abstract

Standard network meta‐analysis (NMA) and indirect comparisons combine aggregate data from multiple studies on treatments of interest, assuming that any effect modifiers are balanced across populations. Population adjustment methods relax this assumption using individual patient data from one or more studies. However, current matching‐adjusted indirect comparison and simulated treatment comparison methods are limited to pairwise indirect comparisons and cannot predict into a specified target population. Existing meta‐regression approaches incur aggregation bias. We propose a new method extending the standard NMA framework. An individual level regression model is defined, and aggregate data are fitted by integrating over the covariate distribution to form the likelihood. Motivated by the complexity of the closed form integration, we propose a general numerical approach using quasi‐Monte‐Carlo integration. Covariate correlation structures are accounted for by using copulas. Crucially for decision making, comparisons may be provided in any target population with a given covariate distribution. We illustrate the method with a network of plaque psoriasis treatments. Estimated population‐average treatment effects are similar across study populations, as differences in the distributions of effect modifiers are small. A better fit is achieved than a random effects NMA, uncertainty is substantially reduced by explaining within‐ and between‐study variation, and estimates are more interpretable.

## 1 Introduction

Health policy and reimbursement decisions require unbiased causal estimates of the relative effectiveness of all relevant treatments or interventions for a given patient population. For example, in England, the National Institute for Health and Care Excellence operates a technology appraisal process in which a company submits evidence on the clinical and cost effectiveness of their treatment compared with other relevant treatments. The gold standard of evidence on relative effectiveness is randomized controlled trials in the population of interest. However, it is rare that all relevant treatments have been compared head to head in a single randomized controlled trial; instead, the evidence base is often comprised of several studies, each comparing a subset of the treatments of interest.

**Figure 1:**
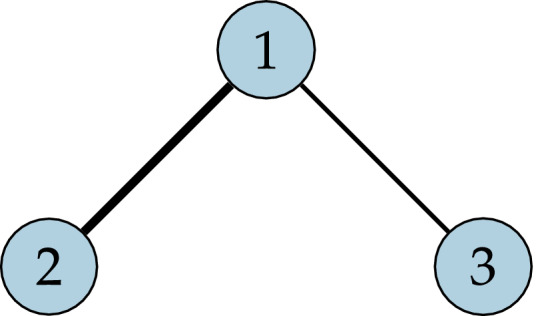
Simple scenario with one 1 *versus* 2 and one 1 *versus* 3 study: individual patient data are available for the 1 *versus* 2 study (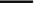); only aggregate data are available for the 1 *versus* 3 study (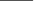); an indirect comparison compares treatments 2 and 3 via the common 1 arm

In the simplest scenario, portrayed in Fig. 1, two treatments of interest (treatments 2 and 3) have been compared separately with a common comparator (treatment 1) in 1 *versus* 2 and 1 *versus* 3 studies. The relative effect of treatment 3 compared with treatment 2 can be estimated by using an indirect comparison *d*_23_=*d*_13_−*d*_12_, where *d*_*ab*_ is the relative effect of *b* compared with *a* on a given scale (Bucher *et al*., 1997). When a larger number of studies and/or treatments are available, a network meta‐analysis (NMA) is the standard approach, of which indirect comparison is a simple special case (Lu and Ades, 2004; Dias, Sutton, Ades and Welton, 2013; Higgins and Whitehead, 1996; Ades, 2003; Lu and Ades, 2006). NMA combines direct evidence (from head‐to‐head studies) and indirect evidence (via a common comparator) in a coherent manner from studies that form a connected network of treatment comparisons. Relative effects between any two treatments *a*,*b* ∈ {2,…,*K*} compared with the reference treatment 1 satisfy the consistency equations *d*_*ab*_=*d*_1*b*_−*d*_1*a*_, enabling any two treatments to be compared even if not included in the same head‐to‐head trial.

Given summary outcomes *y*_·*jk*_ on treatment *k* in study *j*, the standard NMA model can be written as

$$\begin{array}{cc} & y_{\cdot jk}∼\pi_{\mathrm{Agg}}\left(\theta_{\cdot jk}\right),\\ & g\left(\theta_{\cdot jk}\right)=\mu_{j}+\delta_{\mathrm{jk}} \end{array}$$

where *π*_Agg_(·) is a suitable likelihood for the aggregate data. *θ*_·*jk*_ is the expected summary outcome on treatment *k* in study *j*, which is transformed onto the linear predictor scale with a suitable link function *g*(·). The subscript ‘·’ denotes an aggregate level quantity, which will be helpful to distinguish later when introducing individual level data also. *μ*_*j*_ are study‐specific intercepts, and *δ*_*jk*_ is the study‐specific relative effect of treatment *k versus* treatment 1. In a fixed effect NMA, we have *δ*_*jk*_=*d*_1*k*_. We often write *d*_*k*_=*d*_1*k*_ and set *d*_1_=0. In a random‐effects NMA, we instead have *δ*_*jk*_∼*N*(*d*_*k*_,*τ* ^2^), where *τ* ^2^ is the heterogeneity variance (which is assumed common across comparisons), and we set *δ*_*j*1_=*d*_1_=0. Under this parameterization (with treatment 1 as the reference treatment for the entire network), any study with more than one non‐treatment 1 arm has multiple random effects which are given a multivariate normal distribution. Under the assumption that the heterogeneity variance *τ* ^2^ is common across all pairwise comparisons, this has been shown to imply that the correlations between the random effects in each study *j* are corr(*δ*_*ja*_,*δ*_*jb*_)=0.5 for any *a*,*b*>1 (Higgins and Whitehead, 1996). Heterogeneity variances may instead be assumed to be different for each comparison, implying a further set of consistency relationships on the variances and correlations (Lu and Ades, 2009), although in practice a sufficiently rich data structure is seldom available to estimate such a model. We describe models making the common heterogeneity variance assumption throughout this paper, but note that this assumption may be relaxed as previously described (Lu and Ades, 2009). This parameterization is entirely equivalent to the ‘baseline shift’ parameterization of NMA described elsewhere in the literature, where relative effects are defined with respect to a reference arm in each study (e.g. Dias, Sutton, Ades and Welton (2013)), as long as the correlations between random effects are accounted for.

Covariates, whether measured or not, are balanced within each trial by randomization (at least on average) but may differ in distribution between trials. We distinguish between covariates that are effect modifiers, altering the relative effect on a given scale of an active treatment compared with control, and those that are prognostic variables, affecting absolute outcomes on all treatments equally. (Some covariates may be both prognostic and effect modifying.) Standard methods for indirect comparison and NMA require only aggregate data from each trial and make the assumption that the distributions of any effect modifying covariates are balanced between trials so that relative effects are valid across all included study populations—the *constancy of relative effects assumption* (Phillippo *et al*., 2018). When this assumption does not hold, standard indirect comparisons and NMA are subject to bias. If individual patient data (IPD) are available from every study, an IPD network meta‐regression may be performed to adjust for differences in observed effect modifiers and is considered the ‘gold standard’ method (Berlin *et al*., 2002; Lambert *et al*., 2002; Dias, Sutton, Welton and Ades, 2013; Riley *et al*., 2010). In a technology appraisal context, however, such levels of data availability are rare. It is more common for a manufacturer preparing a submission to a reimbursement agency to have IPD on their own studies, but only published aggregate data on their competitors’ studies.

Recently, matching‐adjusted indirect comparison (MAIC) (Signorovitch *et al*., 2010; Ishak *et al*., 2015) and simulated treatment comparison (STC) (Caro and Ishak, 2010; Ishak *et al*., 2015) methods have been proposed that aim to use available IPD in conjunction with aggregate data to adjust for differences in effect modifiers, through reweighting or regression adjustment respectively, to produce population‐adjusted indirect comparisons. These methods rely on the assumption of *conditional constancy of relative effects*, which states that relative effects are constant between populations at any particular level of a set of covariates (the effect modifiers) (Phillippo *et al*., 2018). This is a weaker assumption than constancy of relative effects required for aggregate data NMA. However, these methods are applicable in only the simple two‐study scenario with an IPD study on one treatment of interest and an aggregate data study on the other treatment of interest (possibly sharing a common comparator, as in Fig. 1) and are not generalizable to larger treatment networks. They are also limited to providing a comparison that is adjusted to the population of the trial for which only aggregate data are available, which may not match the target population for the decision.

Meta‐regression methods have also been proposed (Sutton *et al*., 2008; Donegan *et al*., 2013; Jansen, 2012; Saramago *et al*., 2012; Thom *et al*., 2015), which combine available IPD with aggregate data in an NMA framework and estimate interaction effects for effect modifiers. These methods typically assume common regression coefficients at both the individual and the aggregate level, which leads to aggregation bias (a form of ecological bias) when the model is non‐linear (Rothman *et al*., 2008). Two approaches have been proposed to account for this. The first is to split the interaction effect into between‐study (or aggregate level) and within‐study (or individual level) effects (Sutton *et al*., 2008; Donegan *et al*., 2013; Saramago *et al*., 2012; Thom *et al*., 2015). However, in the two‐study scenario (Fig. 1) there are insufficient data to identify the additional between‐study parameter alongside the treatment effect; this approach is therefore not applicable in this scenario. The second approach is to define the aggregate level model by integrating the individual level model over the study population, avoiding aggregation bias by properly relating the two levels. This approach derives from models that are used in the ecological inference literature to incorporate individual level survey or cohort data along with aggregate level data collected by group or area (Jackson *et al*., 2006, 2008). Jansen (2012) first applied such models to combine IPD and aggregate data in NMA; however, so far this approach has been derived for only the simple case of binary outcomes and binary covariates, where the integration reduces to summation.

In this paper, we aim to generalize the ideas of Jansen (2012) to extend to other outcomes and likelihoods and to include continuous covariates. We propose the multilevel network metaregression model ML‐NMR, which is a general method for population‐adjusted indirect comparisons and NMA that can be applied to any connected network of evidence and produce estimates in any specified target population. ML‐NMR has the desirable property that standard IPD and aggregate data NMA are special cases.

To motivate the methods we begin by describing an example of treatments for plaque psoriasis. We then detail ML‐NMR, likelihoods at both the individual and the aggregate level, and a general implementation of ML‐NMR using numerical integration. We illustrate the methods with an example and conclude with a discussion.

Data for the example (including simulated IPD) and code used for the analysis used can be obtained from

https://rss.onlinelibrary.wiley.com/hub/journal/1467985x/series-a-datasets.

### 1.1 Example: plaque psoriasis

**Figure 2:**
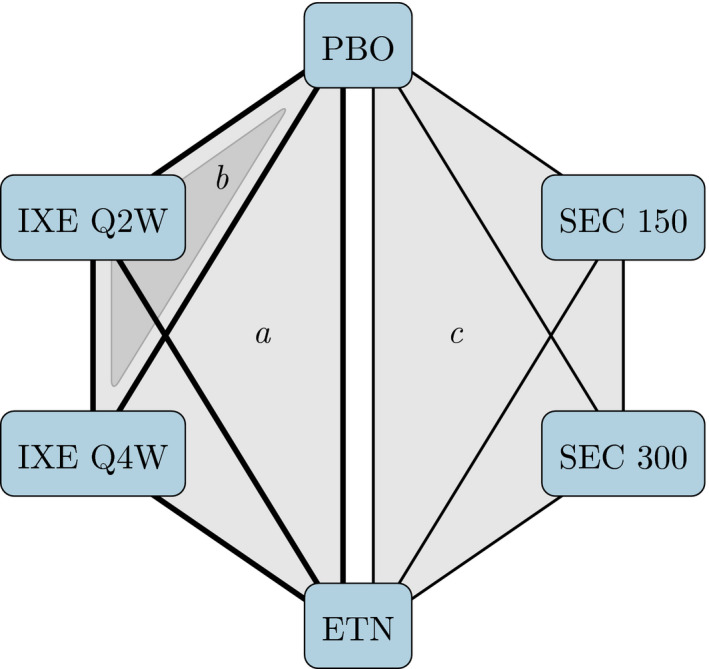
The UNCOVER (Griffiths *et al*., 2015; Gordon *et al*., 2016) and FIXTURE (Langley *et al*., 2014) trials form a network of six treatments: shading indicates comparisons made in *a* the UNCOVER‐2 and UNCOVER‐3 trials, *b* the UNCOVER‐1 trial and *c* the FIXTURE trial (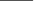, availability of IPD on a comparison; 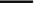, availability of aggregate data on a comparison; PBO, placebo; IXE, ixekizumab; SEC, secukinumab; ETN, etanercept); IXE and SEC were each investigated with two different dosing regimens; the MAIC analysis included only information from the IXE Q2W, SEC 300 and ETN arms, whereas ML‐NMR makes use of all available information

Six treatments for moderate‐to‐severe plaque psoriasis were compared with placebo over 12 weeks in four phase 3 trials. In trials UNCOVER‐1, UNCOVER‐2 and UNCOVER‐3, patients were randomized to receive placebo, etanercept (in UNCOVER‐2 and UNCOVER‐3 only), ixekizumab every 2 weeks, Q2W, or ixekizumab every 4 weeks, Q4W (Griffiths *et al*., 2015; Gordon *et al*., 2016). In the FIXTURE trial, patients were randomized to receive placebo, secukinumab 150 mg, secukinumab 300 mg or etanercept (Langley *et al*., 2014). Fig. 2 displays the resulting treatment network that was formed by the four studies. IPD were available for the three UNCOVER trials. Outcomes of interest include success or failure to achieve 75%, 90% and 100% improvement on the psoriasis area and severity index (PASI) scale (denoted PASI 75, PASI 90 and PASI 100 respectively) at 12 weeks. Information on five clinically relevant covariates that were thought to be potential effect modifiers is available on individuals in the UNCOVER trials, and summary statistics on the same covariates were extracted from the FIXTURE trial (Langley *et al*., 2014). Table 1 summarizes the distribution of these at baseline in each trial.

**Table 1:** Baseline covariate summaries from the UNCOVER and FIXTURE trials†

*Covariate*	*Values for*	*Values for*	*Values for*	*Values for*
	*UNCOVER‐1*	*UNCOVER‐2*	*UNCOVER‐3*	*FIXTURE*
	*(N*=1296*)*	*(N*=1219*)*	*(N*=1339*)*	*(N*=1306*)*
Age, years	45.7 (12.9)	45.0 (13.0)	45.7 (13.1)	44.5 (12.9)
Body surface area, %‡	27.7 (17.3)	26.0 (16.5)	28.3 (17.1)	34.4 (18.9)
Duration of psoriasis, years‡	19.6 (11.9)	18.7 (12.5)	18.2 (12.2)	16.5 (12.0)
Baseline PASI score	20.1 (8.0)	19.6 (7.2)	20.9 (8.2)	23.7 (10.2)
Previous systemic treatment(%) ‡	71.3	64.2	57.1	64.0
Psoriatic arthritis (%)‡	26.3	23.6	20.5	14.7
Male (%)	68.1	67.0	68.2	71.1
Weight, kg‡	92.2 (23.8)	91.6 (22.2)	91.2 (23.5)	83.3 (20.8)

†Reported sample size for the UNCOVER‐2 and UNCOVER‐3 trials after removing two individuals from each study with missing weight. The statistics are the mean (with standard deviation in parentheses) unless otherwise specified. ‡Covariate considered a potential effect modifier, to be included in population adjustment.

A previous MAIC sought to create a population‐adjusted indirect comparison between ixekizumab Q2W and secukinumab 300 mg (the approved dosages), adjusting for these baseline covariates, via the common etanercept arms in UNCOVER‐2 and UNCOVER‐3 and FIXTURE (Strober *et al*., 2016); data from UNCOVER‐1 could not be used, as this study did not include an etanercept arm. Furthermore, comparisons that were made using MAIC are valid only for the population of the study with aggregate data (here FIXTURE) unless further assumptions are made. We shall use ML‐NMR to incorporate all the available data, and produce population‐adjusted indirect comparisons between any pair of treatments in any chosen target population. We shall illustrate this by producing estimates for both the FIXTURE and the UNCOVER study populations, focusing on the PASI 75 outcome. Baseline PASI score and body surface area are highly correlated since the PASI score is based on cut points of body surface area, so we include only body surface area in the adjustment in line with the previous MAIC (Strober *et al*., 2016).

## 2 Multilevel network meta‐regression

Suppose that we wish to compare a set of *K* treatments, using a combination of IPD and aggregate data from a set of *J* trials. The IPD studies provide outcomes *y*_*ijk*_ and covariates $x_{\mathrm{ijk}}$ for each individual *i* in study *j* receiving treatment *k*. The aggregate data studies provide summary outcomes *y*_·*jk*_ for each treatment *k* in study *j*, such as means or event counts. Suppose further that information is available on the joint covariate distribution $f_{\mathrm{jk}}\left(x\right)$ for individuals on treatment *k* in study *j*.

The general framework for ML‐NMR derives from the approach of Jackson *et al*. (2006, 2008), where the aggregate level model is an integration of the individual level model over the population in each trial: individual,

(1a) $$y_{\mathrm{ijk}}∼\pi_{\mathrm{Ind}}\left(\theta_{\mathrm{ijk}}\right),$$

(1b) $$g\left(\theta_{\mathrm{ijk}}\right)=\eta_{\mathrm{jk}}\left(x_{\mathrm{ijk}}\right)=\mu_{j}+x_{\mathrm{ijk}}^{T}\left(\beta_{1}+\beta_{2,\;k}\right)+\gamma_{k};$$

aggregate,

(1c) $$y_{\cdot jk}∼\pi_{\mathrm{Agg}}\left(\theta_{\cdot jk}\right),$$

(1d) $$\theta_{\cdot jk}=\int_{X}g^{-1}\left\{\eta_{\mathrm{jk}}\left(x\right)\right\}f_{\mathrm{jk}}\left(x\right)\;dx.$$

The individual and aggregate level data are given appropriate likelihood distributions *π*_Ind_(·) and *π*_Agg_(·). *θ*_*ijk*_ and $\eta_{\mathrm{jk}}\left(x_{\mathrm{ijk}}\right)$ are the conditional mean outcome and linear predictor for an individual on treatment *k* in trial *j* with covariate vector $x_{\mathrm{ijk}}$. *θ*_·*jk*_ is the marginal mean outcome on treatment *k* in trial *j*. *g*(·) is a suitable link function, and X denotes the support of **x**. The coefficients *μ*_*j*_ are study‐specific baselines, $\beta_{1}$ are coefficients for prognostic variables and $\beta_{2,\;k}$ are coefficients for effect modifiers that are specific to each treatment *k*. The effect of the *k*th treatment (at the individual level), *γ*_*k*_, is defined with respect to the reference treatment 1, and we set *γ*_1_=0 and $\beta_{2,\;1}=0$. Some coefficients in $\beta_{1}$ or $\beta_{2,\;k}$ may be set to 0, if it is known that a particular covariate is not prognostic or effect modifying respectively.

Implementation of the ML‐NMR model (1a), (1b), (1c), (1d) involves two key steps. Firstly, we must derive an aggregate level likelihood *π*_Agg_(·) (1c) from the individual level likelihood *π*_Ind_(·) (1a), ensuring that the relationship between levels is maintained. For several individual level likelihoods the corresponding aggregate likelihood is intuitive, but this is not always so. Secondly, the integral in the aggregate level model (1d) must be evaluated; the form of the derivation depends on the type of covariates (discrete, continuous or a mixture of both) and the link function *g*(·). Simply ‘plugging in’ the mean covariate values leads to aggregation bias whenever the model is non‐linear, since E{g(X)}≠g{E(X)}.

### 2.1 From individual to aggregate likelihoods

The choice of an individual level likelihood distribution is natural; however, care must be taken in subsequently defining the appropriate aggregate level likelihood distribution for the sum (for binary or count data) or average (for continuous data) of the individual outcomes. For example, if a normal individual likelihood $y_{\mathrm{ijk}}∼N\left(\theta_{\mathrm{ijk}},\sigma_{\mathrm{jk}}^{2}\right)$ is used, then the respective aggregate likelihood for the mean of the (independent) individual outcomes will also be normal: $y_{\cdot jk}∼N\left(\theta_{\cdot jk},N_{\mathrm{jk}}^{-1}\sigma_{\mathrm{jk}}^{2}\right)$, where *θ*_·*jk*_ is the mean over the individual *θ*_*ijk*_. Similarly, individual Poisson event counts with likelihood *y*_*ijk*_∼Pois(*θ*_*ijk*_) induce a Poisson aggregate likelihood for the total number of events *y*_·*jk*_∼Pois(*θ*_·*jk*_), where *θ*_·*jk*_ is the sum over the individual *θ*_*ijk*_.

The situation is not so straightforward when binary data are observed, as for our psoriasis example. For each individual, the binary outcome of success or failure to achieve PASI 75 follows a Bernoulli distribution, with some individual success probability *p*_*ijk*_ which is modelled with a probit link function as

(2) $$\begin{array}{cc} & y_{\mathrm{ijk}}∼\text{Bern}\left(p_{\mathrm{ijk}}\right),\\ & p_{\mathrm{ijk}}=\theta_{\mathrm{ijk}}=\Phi \left\{\mu_{j}+x_{\mathrm{ijk}}^{T}\left(\beta_{1}+\beta_{2,\;k}\right)+\gamma_{k}\right\} \end{array}$$

where Φ(·) is the standard normal cumulative distribution function. A probit link was chosen here to remain comparable with previous analyses; another choice is the logit link, although in practice the results will be very similar (Chambers and Cox, 1967).

The total number of events is a sum over individual Bernoulli outcomes, each with different success probabilities due to differences in prognostic factors and effect modifiers, and so the respective aggregate likelihood could be considered approximately binomial,

(3) $$y_{\cdot jk}∼\text{Bin}\left(N_{\mathrm{jk}},{\bar{p}}_{\mathrm{jk}}\right),$$

with an average probability of success ${\bar{p}}_{\mathrm{jk}}$. This approximation works well only when the individual event probabilities *p*_*ijk*_ are all approximately equal for a given study *j* and treatment *k* (Ehm, 1991). The true aggregate likelihood is Poisson binomial, parameterized by a vector $p_{\mathrm{jk}}$ of success probabilities for each individual and has the same mean but a smaller variance than the binomial approximation (3) (Wang, 1993). However, it is not possible to estimate $p_{\mathrm{jk}}$ from the available aggregate data, so we use an approximation (Le Cam, 1960), whereby both parameters of a binomial distribution are adjusted to match both the variance and the mean of the Poisson binomial:

(4) $$y_{\cdot jk}∼\text{Bin}\left(N_{\mathrm{jk}}^{\prime },{\bar{p}}_{\mathrm{jk}}^{\prime }\right),$$

where

$$\begin{array}{cc} N_{\mathrm{jk}}^{\prime } & =\frac{\sum_{i}p_{\mathrm{ijk}}}{{\overset{¯}{p}}_{\mathrm{jk}}^{\prime }}=N_{\mathrm{jk}}\frac{{\overset{¯}{p}}_{\mathrm{jk}}^{2}}{{\overset{¯}{p^{2}}}_{\;\;\;jk}},\\ {\overset{¯}{p}}_{\mathrm{jk}}^{\prime } & =\frac{\sum_{i}p_{\mathrm{ijk}}^{2}}{\sum_{i}p_{\mathrm{ijk}}}=\frac{{\overset{¯}{p{}^{2}}}_{\;\;\;jk}}{{\overset{¯}{p}}_{\mathrm{jk}}} \end{array}$$

defining ${\bar{p^{2}}}_{\;\;\;jk}=N_{\mathrm{jk}}^{-1}\Sigma_{i}p_{\mathrm{ijk}}^{2}$. The mean probability parameter ${\bar{p}}_{\mathrm{jk}}$ is modelled with *θ*_·*jk*_ (equation (1d)) and a probit link:

$${\bar{p}}_{\mathrm{jk}}=\theta_{\cdot jk}=\int_{X}\Phi \left\{\eta_{\mathrm{jk}}\left(x\right)\right\}f_{\mathrm{jk}}\left(x\right)dx.$$

The second parameter ${\bar{p^{2}}}_{\;\;\;jk}$ (which is related to the variance of the individual probabilities) is modelled in a similar manner, where the integration in equation (1d) is over the *squared* probabilities:

$${\bar{p^{2}}}_{\;\;\;jk}=\int_{X}\Phi {\left\{\eta_{\mathrm{jk}}\left(x\right)\right\}}^{2}f_{\mathrm{jk}}\left(x\right)dx.$$

A shifted binomial approximation further matches the skewness of the Poisson binomial distribution by adjusting three parameters (the third being an additional ‘shift’ parameter) (Peköz *et al*., 2009); however, in practice the largest improvements arise from the use of the two‐parameter approximation (4) over the simple approximation (3), with the three‐parameter approximation giving little additional improvement at the expense of greater complexity and computational cost (Peköz *et al*., 2010). We therefore use the two‐parameter approximation in our models.

### 2.2 Deriving the aggregate level model

Implementing ML‐NMR involves an integration step in the aggregate level model (1d). In a Bayesian framework, ML‐NMR is likely to be implemented by using Markov chain Monte Carlo (MCMC) sampling, e.g. in WinBUGS, JAGS or Stan. At each iteration of the MCMC algorithm, we must evaluate integral (1d) at a given set of values for the parameters $\left(\mu_{j},\beta_{1},\beta_{2,\;k},\gamma_{k}\right)$. In a frequentist analysis, integral (1d) is evaluated at every iteration of an estimation routine (e.g. maximizing the likelihood). In this paper, we focus on the Bayesian approach.

If the covariates **x** are all discrete, then integral (1d) becomes a summation which is straightforward to perform:

(5) $$\theta_{\cdot jk}=\sum_{x\in X}g^{-1}\left\{\eta_{\mathrm{jk}}\left(x\right)\right\}f_{\mathrm{jk}}\left(x\right),$$

where now X is the set of discrete levels of **x**, and $f_{\mathrm{jk}}\left(x\right)$ is the proportion of individuals in each level on treatment *k* in the *j*th trial. Jansen (2012) described how a network meta‐analysis combining IPD and aggregate data may be performed by using equation (5) in a logistic regression scenario with only discrete covariates, and using the simple one‐parameter binomial approximation to the Poisson binomial likelihood (equation (3) above).

For continuous covariates, we can use either algebraic or numerical methods to implement ML‐NMR. Analytic approaches to performing the aggregation integral (1d) provide insight into the mathematical nature of the multilevel model (see the on‐line appendix A.1). However, such approaches are context specific, requiring a different approach for each link function, and are of little use when the marginalization integral becomes intractable (most notably when covariates are skewed). There is, therefore, a need for a general numerical approach which is both flexible and sufficiently robust to be widely applicable, regardless of the model form or the distributions of covariates.

#### 2.2.1 A general numerical method

We propose to evaluate the aggregation integral (1d) by using numerical integration at each iteration of the MCMC (or maximum likelihood) algorithm. Quadrature rules are fast and accurate but degrade in higher dimensions (e.g. beyond two or three covariates) and may rely on the specific form of *f*_*jk*_(·) or *g*(·) (such as Gaussian quadrature). Instead, we shall use Monte Carlo integration to evaluate integral (1d).

The integral (1d) over the covariate distribution *f*_*jk*_(·) is approximated by a summation over $\tilde{N}$ values of ${\tilde{x}}_{\mathrm{jk}}∼f_{\mathrm{jk}}\left(\cdot \right)$:

(6) $$\begin{array}{cc} \theta_{\cdot jk} & =\int_{X}g^{-1}\left\{\eta_{\mathrm{jk}}\left(x\right)\right\}f_{\mathrm{jk}}\left(x\right)dx\\ & \approx \frac{1}{\tilde{N}}\sum g^{-1}\left\{\eta_{\mathrm{jk}}\left({\tilde{x}}_{\mathrm{jk}}\right)\right\}. \end{array}$$

For standard Monte Carlo integration, ${\tilde{x}}_{\mathrm{jk}}$ are sampled (pseudo)randomly from the covariate distribution *f*_*jk*_(·); however, this can require large numbers of integration points to obtain sufficiently accurate estimates. The approximation can be made more efficient by using a quasi‐random sample of points (quasi‐Monte‐Carlo sampling (QMC)), which are chosen sequentially to cover the covariate space X more uniformly than pseudorandom samples, resulting in fewer integration points needed to achieve the same accuracy. We obtain a quasi‐random sample of ${\tilde{x}}_{\mathrm{jk}}$ from the covariate distribution *f*_*jk*_(·) by using a Sobol’ sequence (Sobol’, 1967). The Sobol’ sequence generates points in the unit hypercube with one dimension for each covariate, which are then transformed to the required distribution by using the inverse cumulative distribution function method. The integration points ${\tilde{x}}_{\mathrm{jk}}$ are treated as fixed inputs for the MCMC run and used to evaluate sum (6) at each iteration (i.e. at each posterior sample of the model parameters). QMC integration may achieve integration error rates of the order ${\tilde{N}}^{-1}$, compared with standard Monte Carlo sampling which achieves only ${\tilde{N}}^{-1/2}$ (Niederreiter, 1978; Caflisch, 1998). In other words, when using 10000 integration points drawn from a Sobol’ sequence, we would expect accuracy down to around four decimal places, compared with an accuracy of two decimal places by using standard Monte Carlo sampling.

The goodness of fit may be assessed by using the residual deviance. For binary data, calculation of the residual deviance for the individual level Bernoulli likelihood is straightforward; for the aggregate level likelihood we again use a binomial approximation for tractability.

We implement this numerical approach in a Bayesian framework by using R (R Core Team, 2017) and Stan (Carpenter *et al*., 2017); full general purpose code is available in the on‐line supplementary materials for the probit regression case, which can be adapted to other likelihoods and link functions. The code makes use of *QR*‐decomposition and centring which make computation highly efficient. The computation time is driven by the amount of IPD that is available and the number of integration points chosen and is not markedly increased over an equivalent full IPD network meta‐regression. For example, the ML‐NMR model for the plaque psoriasis example takes around 5 min to run on a modern desktop computer.

#### 2.2.2 Using published marginal covariate information

When using ML‐NMR in practice, we typically have access to only limited covariate information from the aggregate data studies. This is often in the form of published marginal covariate summaries, and we have no information on the correlation structure between the covariates or on the distributional form of the marginal distributions. Where the forms of the marginal covariate distributions in the aggregate data studies are unknown, we can instead choose distributional forms for these covariates on the basis of their theoretical properties and/or to match approximately the observed distributional forms in the IPD studies. For example, a covariate may be well known to be approximately log‐normal and/or observed as such in the IPD, and we can then assume a marginal distribution for this covariate in the aggregate data studies to match the published summary statistics. To account for the missing correlation structure, rather than assuming that all correlations are 0 (which may be unreasonable), we can utilize the correlation structure that is observed among the covariates in the IPD studies and assume that this holds for the covariates in the aggregate data studies. It seems reasonable to assume that, although the marginal summaries for each covariate may change from study to study (e.g. the proportion of males, or mean and standard deviation of weight), the relationships between covariates are likely to remain similar (for example the duration of disease is positively correlated with the number of previous lines of treatment). We encode this assumption in the joint covariate distributions for the aggregate data studies by using a Gaussian copula (Nelsen, 2006), so that the correlation matrix of the covariates in the aggregate data studies match those of the IPD studies, while retaining the given marginal distributions (see the on‐line appendix A.3).

### 2.3 Using the shared effect modifier assumption

The data requirements for estimating a treatment effect and independent effect modifier interaction terms for a given treatment *k* are either

(a) IPD from one or more trials including treatment *k* or
(b) sufficiently many aggregate data studies including treatment *k*, with enough variation in covariate values (equivalent to the requirements of a standard aggregate data meta‐regression).

In the case that neither of these requirements can be met, either informative prior distributions or additional assumptions are required to estimate the model.

The shared effect modifier assumption (Phillippo *et al*., 2016, 2018) can make the model estimable by assuming that the effect modifier interaction coefficients $\beta_{2,\;k}$ are identical among a set or class of treatments k∈T. The data requirements that were described above then apply to the set T as a whole, rather than to each individual treatment.

To relax the shared effect modifier assumption for a set of treatments T, we can instead assume that the interactions are exchangeable. A hierarchical model is placed on the interaction coefficients

(7) $$\beta_{2,\;k;l}∼N\left(m_{\beta_{2},\;l},\sigma_{\beta_{2},\;l}^{2}\right)$$

for k∈T and each coefficient $\beta_{2,\;k;l}$ in the vector $\beta_{2,\;k}$, where *l* indexes the various covariates. The hyperparameters $m_{\beta_{2},\;l}$ and $\sigma_{\beta_{2},\;l}^{2}$ are themselves given prior distributions in a Bayesian framework. There may be multiple (mutually exclusive) treatment sets $T_{1},T_{2},\ldots$ within the treatment network, and some of these may contain only a single treatment on its own. Different assumptions regarding the effect modifier interactions may be made within each set if required.

In practice, the data requirements for estimating exchangeable interactions (7) may also be beyond the data that are available. In particular, the estimation of the variance components $\sigma_{\beta_{2},\;l}^{2}$ is challenging and improves only with increasing numbers of treatments in T and/or informative prior distributions. If there are multiple treatment sets within the network for which the shared effect modifier assumption is made, then the variance components could be assumed equal between the treatment sets to aid estimation further.

### 2.4 Assessing residual heterogeneity and inconsistency

The key assumption of population adjustment methods is that relative effects are constant given the effect modifiers adjusted for (conditional constancy of relative effects; see Phillippo *et al*. (2016, 2018)). Within the ML‐NMR framework, this assumption corresponds to the individual level relative effects *γ*_*k*_ being constant across populations—in other words, we fit a fixed effect model. In the two‐study scenario (Fig. 1) this is an untestable assumption. However, with a larger network of studies and treatments it is possible to assess the conditional constancy of relative effects assumption.

A random‐effects model may be fitted to check for any residual heterogeneity remaining after adjusting for the given effect modifiers. This is achieved by modifying the linear predictor $\eta_{\mathrm{jk}}\left(x\right)$ in equation (1a), (1b), (1c), (1d) as follows: fixed effect,

(8) $$\eta_{\mathrm{jk}}\left(x\right)=\mu_{j}+x^{T}\left(\beta_{1}+\beta_{2,\;k}\right)+\delta_{\mathrm{jk}},\delta_{\mathrm{jk}}=\gamma_{k};$$

random effects,

(9a) $$\eta_{\mathrm{jk}}\left(x\right)=\mu_{j}+x^{T}\left(\beta_{1}+\beta_{2,\;k}\right)+\delta_{\mathrm{jk}},$$

(9b) $$\delta_{\mathrm{jk}}∼N\left(\gamma_{k},\tau^{2}\right),$$

(9c) $$\text{corr}(\delta_{\mathrm{ja}},\delta_{\mathrm{jb}})=0.5$$

where *δ*_*j*1_=0. The random effects are multivariate normal with marginal distributions given by expression (9b) and correlations equal to 0.5, equation (9c), under the assumption of common heterogeneity variance *τ* ^2^ (Higgins and Whitehead, 1996). For two‐arm studies against treatment 1, there is a single univariate normal random effect on the non‐treatment 1 arm, with distribution given by expression (9b). The appropriateness of the conditional constancy of relative effects assumption can then be assessed by comparing model fit (e.g. by using the deviance information criterion; Spiegelhalter *et al*. (2002)) between the fixed and random‐effects models and by examining the posterior distribution of the residual heterogeneity variance *τ* ^2^. The random‐effects model (9a), (9b), (9c) is likely to be more widely applicable in practice than the exchangeable interactions model (7) as a relaxation of the standard ML‐NMR model (1a), (1b), (1c), (1d), since the data requirements are lesser.

For ML‐NMR, consistency applies to both the individual level treatment effects and the effect modifier interactions (as described for meta‐regression by Donegan *et al*. (2017)):

(10a) $$\gamma_{\mathrm{ab}}=\gamma_{b}-\gamma_{a},$$

(10b) $$\beta_{2,\;ab}=\beta_{2,\;b}-\beta_{2,\;a}.$$

Standard approaches for investigating inconsistency such as unrelated mean effects models (Dias, Welton, Sutton, Caldwell, Lu and Ades, 2011) and node splitting models (Dias *et al*., 2010) can also be applied in the context of ML‐NMR. We describe application of these approaches to ML‐NMR models in the on‐line appendix A.4.

The presence of residual heterogeneity or inconsistency has several potential causes. For example, there may be effect modifiers that have not been included in the model or other model misspecification, the assumed joint covariate distributions used to adjust the results from aggregate studies may be incorrect or the shared effect modifier assumption (if it was used) may be invalid. Attempts may be made to rectify these issues in a revised model—if data permit—and the residual heterogeneity and inconsistency of the revised model may then be checked.

### 2.5 Producing estimates for a specific target population

To be relevant for decision making, population‐adjusted estimates must be produced for the relevant target population. ML‐NMR can produce population‐adjusted estimates of quantities of interest in any target population for which covariate information is available. For example, the quantities of interest may be average treatment effects (contrasts), or the proportion of individuals achieving PASI 75 response. In general, we consider estimating the average ${\bar{h}}_{\left(P\right)}$ of a quantity *h*(**x**,***ξ***) in a target population *P*, which is some function of the covariates **x** and a set ***ξ*** of the parameters *μ*_(*P*)_, $\beta_{1}$, $\beta_{2,\;k}$ and *γ*_*k*_ (with the required parameters depending on the quantity of interest). As a function of the parameters, ${\bar{h}}_{\left(P\right)}$ has a posterior distribution; when using MCMC sampling, ${\bar{h}}_{\left(P\right)}$ is evaluated at each iteration to obtain posterior samples.

Given the joint covariate distribution *f*_(*P*)_(·) in the target population, ${\bar{h}}_{\left(P\right)}$ is obtained by integrating over the joint covariate distribution (as a generalization of equation (1d)):

(11) $${\bar{h}}_{\left(P\right)}=\int_{X}h\left(x,\xi \right)f_{\left(P\right)}\left(x\right)dx.$$

The integral may be evaluated by using QMC integration, as described above.

Alternatively, if the target population is represented by a study or registry with individual covariate information available, ${\bar{h}}_{\left(P\right)}$ is obtained by taking the average of *h*(**x**,***ξ***) applied to each individual in *P*:

(12) $${\bar{h}}_{\left(P\right)}=\frac{1}{N_{P}}\sum_{i=1}^{N_{P}}h\left(x_{i},\xi \right),$$

where $N_{P}$ is the number of individuals in the sample, each with covariate information $x_{i}$.

To calculate the population‐average relative effect *d*_*ab*(*P*)_ between treatments *b* and *a*, we define

(13) $$h\left(x,\beta_{2,\;a},\beta_{2,\;b},\gamma_{a},\gamma_{b}\right)=x^{T}\left(\beta_{2,\;b}-\beta_{2,\;a}\right)+\gamma_{b}-\gamma_{a}.$$

In this case, since the population‐average relative effects are given on the transformed linear predictor scale, both equation (12) and equation (11) are equivalent to ‘plugging‐in’ mean covariate values: ${\bar{h}}_{\left(P\right)}={\bar{x}}^{T}\left(\beta_{2,\;b}-\beta_{2,\;a}\right)+\gamma_{b}-\gamma_{a}$. Equation (13) makes use of the consistency equations: the individual relative effects for *b versus a* are *γ*_*ab*_=*γ*_*b*_−*γ*_*a*_, with effect modifier coefficients $\beta_{2,\;ab}=\beta_{2,\;b}-\beta_{2,\;a}$. These consistency equations apply at the individual level and imply consistency of the population‐average relative effects within each population, *d*_*ab*(*P*)_=*d*_*b*(*P*)_−*d*_*a*(*P*)_, but *not* across populations. Standard NMA is a special case where there is no effect modification (or effect modifiers are balanced between all studies), so the population‐average relative effects are identical between studies and the usual NMA consistency equations *d*_*ab*_=*d*_*b*_−*d*_*a*_ are recovered.

To calculate the predicted proportion of individuals achieving PASI 75 response on treatment *k* in population *P*, we define

(14) $$h\left(x,\mu_{\left(P\right)},\beta_{1},\beta_{2,\;k},\gamma_{k}\right)=\Phi \left\{\mu_{\left(P\right)}+x^{T}\left(\beta_{1}+\beta_{2,\;k}\right)+\gamma_{k}\right\}$$

where *μ*_(*P*)_ is the individual level reference effect in population *P*, which may be equal to *μ*_*j*_ if *P* is study *j* in the analysis, or may be estimated from external data on *P*.

## 3 Application to plaque psoriasis example

To implement the aggregate level model, we use the QMC integration approach that was described above to integrate the individual level model (1b) over the covariate distribution in the FIXTURE trial (Table 1). First, 10000 points are taken from a five‐dimensional Sobol’ sequence, one dimension for each covariate: body surface area, duration of psoriasis, previous systemic treatment, psoriatic arthritis and weight. Marginal distributions for each covariate in the FIXTURE trial are chosen to match the summary statistics reported, with specific form based on theoretical properties and the observed distributions in the UNCOVER trials: weight and duration are given a gamma distribution to account for skewness, and body surface area as a percentage is given a scaled logit–normal distribution. Previous systemic treatment and psoriatic arthritis are binary covariates. Since no information on the correlations between covariates is available in the FIXTURE trial, these are assumed to match those observed in the UNCOVER trials. To account for this, we compute a correlation matrix for the UNCOVER trials and impose this on the Sobol’ points by using a Gaussian copula, before transforming to the required marginal distributions by using the inverse cumulative distribution function method (see the on‐line appendix A.3). The resulting integration points capture the correlations between the covariates (for example a longer duration of psoriasis is correlated with having previous systemic treatment) while preserving the marginal distribution for each covariate. Figs A.1 and A.2 in appendix A.5 demonstrate that empirical integration error rates of the order ${\tilde{N}}^{-1}$ are indeed achieved over the entire posterior distribution of the parameters, for both $\bar{p}$ and $\bar{p^{2}}$ in each arm of the FIXTURE study.

We then fit an ML‐NMR model to the PASI 75 outcomes, including interaction terms for the five potential effect modifiers in Table 1. We take a Bayesian approach implemented in Stan (Carpenter *et al*., 2017), placing a non‐informative *N*(0,100^2^) prior distribution on each parameter. A probit link function is used, as the binary outcome is a dichotomization of the continuous underlying PASI scale, which means that the model parameters and treatment effects are interpreted as standardized mean differences (SMDs) on the PASI scale. With only two aggregate data secukinumab treatment arms available, it is not possible to identify a model with five distinct effect modifier interactions and a treatment effect for each secukinumab dose. However, since secukinumab and ixekizumab share modes of action as interleukin‐17A blockers, we assume that the effect modifier interaction parameters are common between these treatments across all doses (the shared effect modifier assumption) to identify the model. Four parallel chains were run for 2000 iterations each (the first 1000 iterations were discarded as warm‐up). Convergence was assessed by using the potential scale reduction factor $\hat{R}$ (Gelman *et al*. (2013), pages 284–285), and effective sample sizes and Monte Carlo standard errors guided the choice of the number of iterations. There were no divergent transitions.

Finally, we produce estimates of contrasts between each pair of treatments, and of the proportion of individuals achieving PASI 75, in both the UNCOVER and the FIXTURE trial populations. For the UNCOVER populations, these are produced by averaging the predicted SMD contrast on each pair of treatments (13), or the predicted proportion achieving PASI 75 (14), over every individual by using equation (12). For the FIXTURE population, these are produced by integrating the respective quantities over the joint covariate distribution by using numerical integration as in equation (11).

The resulting contrast estimates are shown in Table 2 (see also Fig. A.3 in the on‐line appendix A.5). There are small differences in the estimated average treatment effects in each population; for example, etanercept appears slightly more effective relative to placebo in the FIXTURE study population than in the UNCOVER study populations. Examining the estimated effect modifier interactions in Table 3 alongside the covariate summaries in Table 1, we see that this is to be expected: the differences in mean covariate values between study populations are small when combined with the size of the interaction terms. Furthermore, a random‐effects NMA of the studies estimates the between‐study heterogeneity standard deviation to be 0.17 (0.02, 0.46), which is moderate compared with the size of the average treatment effects. Using MAIC, the comparison between ixekizumab Q2W and secukinumab 300 mg in the FIXTURE population is estimated as an SMD of 0.28 (0.00, 0.56) in favour of ixekizumab Q2W; with ML‐NMR, we estimate 0.34 (0.10, 0.58). (A standard indirect comparison estimates 0.37 (0.12, 0.63).) The point estimates are similar between the two population adjustment approaches, as we would expect, but ML‐NMR has reduced uncertainty compared with the MAIC method because of incorporating all available information from the studies. The random‐effects NMA estimate for this contrast is 0.45 (−0.02, 0.92), which assumes that any imbalance in effect modifiers is random. Since the differences in effect modifiers between trials are small, the possible bias in the random‐effects NMA estimate is likely to be small also; however, ML‐NMR increases the precision of the estimates by explaining the within‐trial variation due to effect modification.

**Table 2:** Estimated SMD contrasts and 95% credible intervals for each pair of treatments in each study population (for ML‐NMR) and from a random‐effects NMA†

*Contrast*	*Results for ML‐NMR study population*				*Results for random‐*
					*effects NMA*
	*FIXTURE*	*UNCOVER‐1*	*UNCOVER‐2*	*UNCOVER‐3*	
IXE Q2W *versus*	2.94	2.98	2.95	2.93	2.91
	(2.74, 3.14)	(2.80, 3.17)	(2.77, 3.13)	(2.76, 3.11)	(2.64, 3.19)
IXE Q4W *versus*	2.65	2.69	2.66	2.64	2.61
	(2.45, 2.84)	(2.51, 2.89)	(2.47, 2.84)	(2.46, 2.82)	(2.32, 2.88)
ETN *versus* PBO	1.74	1.65	1.64	1.65	1.61
	(1.55, 1.93)	(1.47, 1.83)	(1.46, 1.81)	(1.47, 1.81)	(1.34, 1.91)
SEC 150 *versus*	2.29	2.33	2.30	2.28	2.16
	(2.07, 2.53)	(2.10, 2.58)	(2.07, 2.54)	(2.05, 2.52)	(1.71, 2.61)
SEC 300 *versus*	2.60	2.64	2.61	2.59	2.46
	(2.36, 2.83)	(2.40, 2.90)	(2.36, 2.86)	(2.35, 2.83)	(2.02, 2.89)
IXE Q4W *versus*	−0.30	−0.30	−0.30	−0.30	−0.31
	(−0.42, −0.17)	(−0.42, −0.17)	(−0.42, −0.17)	(−0.42, −0.17)	(−0.57, −0.05)
ETN *versus*	−1.20‡	−1.33	−1.31	−1.29	−1.30
	(−1.35, −1.06)	(−1.47, −1.19)	(−1.45, −1.18)	(−1.42, −1.15)	(−1.58, −1.01)
SEC 150 *versus*	−0.65	−0.65	−0.65	−0.65	−0.75
	(−0.89, −0.42)	(−0.89, −0.42)	(−0.89, −0.42)	(−0.89, −0.42)	(−1.24, −0.25)
SEC 300 *versus*	−0.34§	−0.34	−0.34	−0.34	−0.45
	(−0.58, −0.10)	(−0.58, −0.10)	(−0.58, −0.10)	(−0.58, −0.10)	(−0.92, 0.02)
ETN *versus*	−0.91	−1.04	−1.02	−0.99	−0.99
	(−1.05, −0.75)	(−1.17, −0.90)	(−1.15, −0.89)	(−1.12, −0.85)	(−1.27, −0.68)
SEC 150 *versus*	−0.36	−0.36	−0.36	−0.36	−0.45
	(−0.59, −0.12)	(−0.59, −0.12)	(−0.59, −0.12)	(−0.59, −0.12)	(−0.93, 0.03)
SEC 300 *versus*	−0.05	−0.05	−0.05	−0.05	−0.14
	(−0.27, 0.19)	(−0.27, 0.19)	(−0.27, 0.19)	(−0.27, 0.19)	(−0.62, 0.31)
SEC 150 *versus*	0.55	0.68	0.66	0.63	0.54
	(0.36, 0.74)	(0.48, 0.88)	(0.46, 0.87)	(0.43, 0.84)	(0.11, 0.97)
SEC 300 *versus*	0.86	0.99	0.97	0.94	0.85
	(0.65, 1.06)	(0.77, 1.20)	(0.75, 1.18)	(0.72, 1.14)	(0.41, 1.26)
SEC 300 *versus*	0.31	0.31	0.31	0.31	0.31
	(0.11, 0.53)	(0.11, 0.53)	(0.11, 0.53)	(0.11, 0.53)	(−0.16, 0.77)

†See the caption for Fig. 2 for definitions of the terms in the contrasts. The ML‐NMR contrast estimates between ixekizumab and secukinumab treatments are the same in every population because of the shared effect modifier assumption for these treatments. ‡The MAIC estimate is −1.18 (−1.37, −0.99). A standard indirect comparison uses the pooled study estimate −1.27 (−1.42, −1.12) from the UNCOVER‐2 and UNCOVER‐3 trials. §The MAIC estimate is −0.28 (−0.56, −0.00). The standard indirect comparison estimate is −0.37 (−0.63, 0.12).

**Table 3:** Estimated interactions for each treatment class and potential effect modifier, and estimated individual level treatment effects†

	*Results for the following treatment classes:*	
	*Anti‐TNFα*	*IL‐17A blocker*
Effect modifier interaction		
Previous systemic use	−0.00 (−0.38, 0.35)	0.12 (−0.21, 0.46)
Duration of psoriasis, per 10 years	0.14 (−0.03, 0.30)	0.17 (0.02, 0.33)
Body surface area, per 10%	0.06 (−0.05, 0.17)	0.02 (−0.09, 0.13)
Weight, per 10 kg	−0.10 (−0.18, −0.02)	−0.04 (−0.11, 0.04)
Psoriatic arthritis	0.01 (−0.43, 0.48)	0.25 (−0.17, 0.71)
Reference individual treatment effect		
IXE Q2W		2.82 (2.56, 3.10)
IXE Q4W		2.52 (2.25, 2.80)
ETN	1.67 (1.38, 1.96)	
SEC 150		2.16 (1.86, 2.49)
SEC 300		2.47 (2.17, 2.79)

†All estimates are standardized mean differences *versus* placebo, with 95% credible intervals.

**Figure 3:**
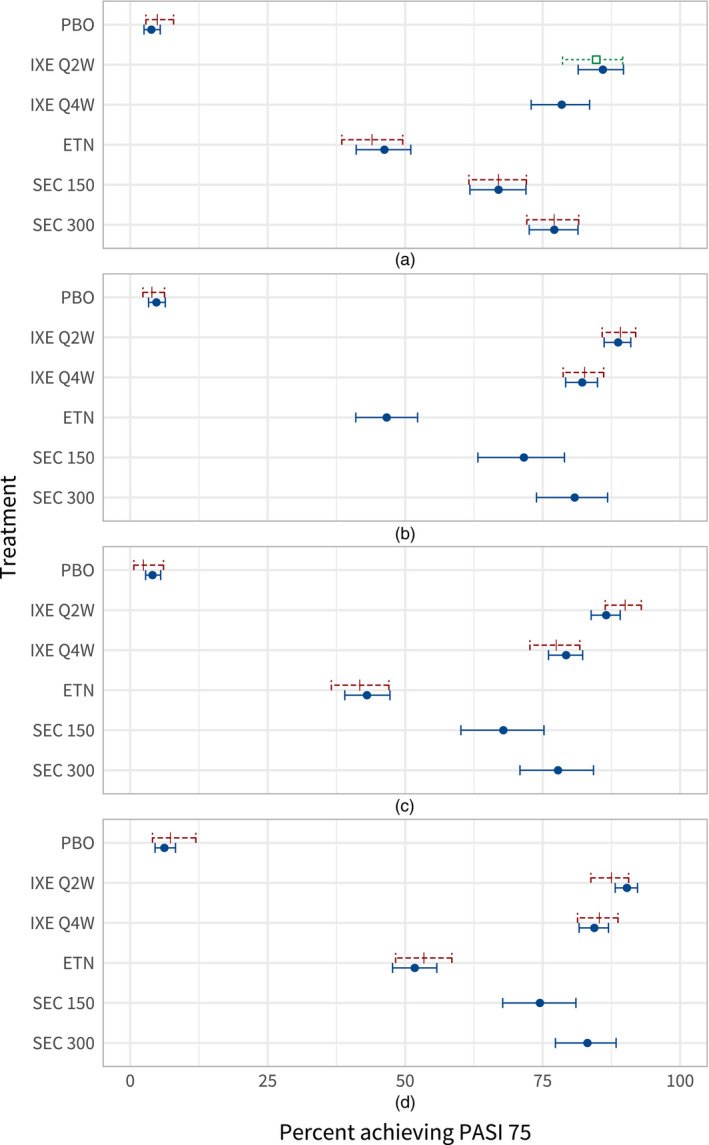
Estimated proportion of individuals achieving PASI 75 on each treatment, in each study population (the MAIC estimate is produced in the FIXTURE study population, and the corresponding interval is a 95% confidence interval as MAIC is a frequentist method) (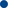, ML‐NMR; 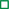, MAIC; 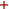, observed): (a) FIXTURE; (b) UNCOVER‐1; (c) UNCOVER‐2; (d) UNCOVER‐3

Fig. 3 shows the estimated proportion of individuals achieving PASI 75 in each population, using MAIC and ML‐NMR (see also Table A.1 in the on‐line appendix A.5). Again, ML‐NMR has reduced uncertainty compared with the MAIC method, and we can produce estimates for any target population—not just the FIXTURE trial population.

Every active treatment is effective compared with placebo, with the class of interleukin 17A blockers more effective than anti‐TNF*α* treatment. Ixekizumab Q2W displays the highest estimated proportion of individuals achieving PASI 75, with posterior mean estimates ranging from 85.9% to 90.3% across the UNCOVER and FIXTURE studies. The 95% credible intervals for comparisons of ixekizumab Q2W against every other treatment exclude zero (on the probit SMD scale), in all the study populations that were assessed. In a decision‐making context, estimates could be produced for the decision target population with a defined covariate distribution, which need not match any of the FIXTURE or UNCOVER studies.

The total residual deviance was 3146.26 on 3858 data points, of which the contribution from 3854 individual data points was 3141.81, and 4.45 from four aggregate data points, demonstrating that the model is a good fit to both the IPD and aggregate data. Standard fixed and random‐effects NMA models have a total residual deviance of 3216.01 and 3210.44 respectively. Comparing the deviance information criterion values (Spiegelhalter *et al*., 2002) between the various approaches, ML‐NMR has a lower value (3170) than either fixed (3225) or random‐effects (3223) NMA. The ML‐NMR model achieves a better fit than the random‐effects NMA and is more interpretable and informative, as the heterogeneity is explained rather than averaged over.

## 4 Discussion

In this paper, we have proposed a new method for population‐adjusted indirect comparisons and network meta‐regression. ML‐NMR derives from a method that was presented in the ecological inference literature, where the aggregate level model is obtained by integrating the individual level model over the covariate distribution (Jackson *et al*., 2006, 2008). The methods in the ecological inference literature have been applied in the context of NMA previously but were derived only for the simple case of a binary outcome and binary covariates (Jansen, 2012). There are several key advantages to this approach, particularly in comparison with methods such as the MAIC (Signorovitch *et al*., 2010; Ishak *et al*., 2015), STC (Caro and Ishak, 2010; Ishak *et al*., 2015) or network meta‐regression based approaches (Sutton *et al*., 2008; Donegan *et al*., 2013; Saramago *et al*., 2012; Thom *et al*., 2015). ML‐NMR is applicable to treatment networks of any size, enabling use of all available information, unlike the MAIC or STC methods. There is no need to split the estimation of interaction effects as aggregation bias is inherently avoided by design; the model therefore remains identifiable even in the two‐study scenario, unlike other meta‐regression approaches. As is crucial for relevance in decision making, comparisons may be provided in any target population given sufficient information on the covariate distribution, without the need for additional assumptions that are required by methods such as the MAIC or STC methods. The target population need not be a clinical trial and could be taken from other sources of data such as registries. Furthermore, using the numerical approach we can easily incorporate correlations between the covariates in the aggregate data imputed from the IPD, with copulas.

Several researchers (Saramago *et al*., 2012; Donegan *et al*., 2013; Riley and Steyerberg, 2010; Riley *et al*., 2008) have used a meta‐regression model with effect modifier interactions split into between‐study terms (i.e. interaction with the mean covariate values in each study) and within‐study terms (i.e. interaction with individual differences from the mean), where the aggregate data studies contribute to the between‐study interaction only. The reason is to avoid ecological bias, focusing interpretation on the within‐study interactions. However, the ecological bias has two potential sources in the context of randomized controlled trials (Greenland, 1992): unobserved effect modifiers, and plugging in mean covariate values into a model with a non‐linear link function (that we refer to as aggregation bias). ML‐NMR avoids the latter since the aggregate level model is appropriately related to the individual level model through integration over the covariate distribution in the aggregate data studies. However, unobserved effect modifiers are still a concern—whether these are individual level covariates, or study level differences in treatment regimens or outcome definitions for example. We have not split the interaction terms in the ML‐NMR model: we assume that there are no unobserved effect modifiers so the conditional constancy of relative effects assumption holds, which is required to produce unbiased estimates of population‐adjusted relative effects (which are the focus of decision making in technology appraisal, rather than estimates of interaction). The ML‐NMR model could be modified to include split interaction terms and to assess the conditional constancy of relative effects assumption in this manner; however, this results in a non‐identifiable model in the two‐study scenario, and probably requires substantial amounts of data to estimate well. Instead, we have proposed assessing the conditional constancy of relative effects assumption by investigating residual heterogeneity and inconsistency (Section 2.4), which is less data intensive.

A previous MAIC analysis based on the UNCOVER‐2, UNCOVER‐3 and FIXTURE trials produced an indirect comparison between ixekizumab Q2W and secukinumab 300 mg, via common etanercept arms (Strober *et al*., 2016). The results of this MAIC are not valid for the FIXTURE population unless the shared effect modifier assumption is made, which leads analysts to arguing that their competitor's trial population is more relevant for the treatment decision at hand (Phillippo *et al*., 2016, 2018). We have had to make the shared effect modifier assumption (within each treatment class) in our analysis to identify the ML‐NMR model, which is probably reasonable, but with more data it would be possible to relax this assumption. Comparisons between other pairs of treatments could also be formed by using MAIC, either via etanercept or via placebo arms, and, in the latter case, data from the UNCOVER‐1 trial could also be included. However, repeated pairwise application of MAIC is unlikely to result in a set of consistent treatment effect estimates—in the same way that multiple pairwise meta‐analyses are unlikely to provide a set of consistent estimates (Caldwell *et al*., 2005). Using ML‐NMR we obtain a full set of consistent relative effects between each pair of treatments, which are also more precise than those from MAIC or from a random‐effects NMA. We have also produced absolute response rates on each treatment, and other effect measures (such as odds ratios) are straightforward to derive. For the psoriasis example, the effect modifier interaction estimates are small compared with the size of the treatment effects, with only duration of psoriasis and weight showing any evidence of moderate interaction. There is also little imbalance between the study populations—the variation in potential effect modifiers within each study is much greater. Consequently, the population‐average relative effect estimates do not differ markedly from the random‐effects NMA. ML‐NMR has, however, substantially reduced the uncertainty by explaining within‐ and between‐study variation.

We conceptualize ML‐NMR as an extension of the standard NMA framework, which is an established and accepted method with a broad literature. Standard IPD and aggregate data NMA are special cases of ML‐NMR, where either all studies or no studies have IPD available respectively. When implemented in a Bayesian framework ML‐NMR retains the flexibility and extensibility of Bayesian NMA, so that, for example, prior information could be utilized, several data types with differing likelihoods could be included or the analysis embedded in a probabilistic cost‐effectiveness framework as widely used by decision makers (Dias, Sutton, Welton and Ades, 2013).

Motivated by the application to plaque psoriasis, we have presented algebraic derivations of the aggregate level model for the probit link function (on‐line appendix A.1). It is possible to derive results for other link functions including the logit (via approximation to the probit, as outlined in appendix A.2) and log‐link (via moment‐generating functions, as in the ecological inference literature (Salway and Wakefield, 2005)). However, these algebraic approaches quickly become complex, are context specific and are not always tractable (e.g. with skew covariates). The general numerical approach that we propose here is straightforward and broadly applicable, and the code that is provided in the supplementary material may be reused with minimal modification. In practical terms, the numerical approach is much quicker to implement than an algebraic approach, and sampling is highly efficient because of optimizations such as *QR*‐decomposition.

All population adjustment methods rely on the availability of covariate information in all the studies included. In a connected network, adjustment is only required for effect modifiers (Phillippo *et al*., 2018) which, being of high clinical relevance, are more likely to be widely reported in publications. Aside from unmeasured or unreported covariates, other forms of missing information are also an issue.

Firstly, it is unlikely for publications to report the correlation structure between covariates, although this may be available on request. Methods such as the MAIC method ignore the correlations between covariates. However, as we have shown algebraically for multivariate normal covariates (on‐line appendix A.1), correlations involving effect modifiers are implicated in aggregation bias along with within‐study variation. Assuming that correlation structures are the same within the aggregate data and IPD, which may be more reasonable than assuming that all correlations are zero, we can impute the missing correlations when generating the aggregate data integration points. Correlation structures could differ between the populations if, for example, different characteristics coexist, or the sampling and randomization methods differ between the trials. However, in our experience varying the assumed correlation structure of the aggregate data trial has negligible effect on the results. We propose a very flexible approach based on copulas, allowing any set of desired marginal distributions to be combined under a given joint correlation structure. For the psoriasis example, we used a simple Gaussian copula. This accounts for linear correlation in the underlying relationships between covariates, but no restrictions are placed on the form of the marginal distributions. For example, skew covariates may be given gamma or log‐normal marginal distributions, and binary covariates are given Bernoulli marginal distributions. Correlations with binary covariates can be incorporated by using Spearman's rank correlation. In practice, we find that this is a very flexible approach and can account for a wide range of observed relationships between covariates; however, other copulas could be used to assert covariance structures under different assumptions (Nelsen, 2006).

Secondly, the true marginal distributions of the aggregate covariates are unknown. In our analysis, we chose distributional forms for the aggregate covariates based on theoretical properties and to match approximately the observed distributional forms in the IPD. However, the true marginal distributions could differ between the populations if, for example, the sampling and randomization methods differ between the trials. In our experience, altering the assumed marginal distributions for the aggregate data trial has little effect on the results.

Finally, missing values are likely to be encountered within the IPD and aggregate data studies. Within the UNCOVER trials, only baseline weight had any missing values. Since the proportion of missing values was so low (0.1%), we simply removed those individuals with missing weight from the analysis. In general, this is not recommended, as complete‐case analyses can incur bias and loss of precision. Multiple imputation is a widely used method of dealing with missing data (Little and Rubin, 2002). The Bayesian framework is well suited to implementing multiple imputation as covariates can be imputed at every iteration of the MCMC sampler, incorporating the uncertainty arising from the missing values in the posterior distribution (Mason *et al*., 2012; Jackson *et al*., 2009). Such approaches have previously been described for IPD NMA (Quartagno and Carpenter, 2016) and apply similarly to ML‐NMR for the studies with IPD by extension. When aggregate data studies have individuals with missing values there may be little that can be done, other than assuming that the summary statistics would be unchanged if the missing values had been observed. Methods for dealing with other issues arising in the analysis of IPD such as non‐compliance (Imbens and Rubin, 1997; DiazOrdaz *et al*., 2018) may also be extended to ML‐NMR for the studies with IPD. Again, there may be little that can be done for aggregate data studies with such issues if a suitable analysis has not been reported in publications.

We have presented a novel approach to analysing treatment networks with mixtures of IPD and aggregate data studies, and examined the properties of such analyses with algebraic derivations. A numerical implementation using QMC integration is both highly efficient and widely applicable. The use of quasi‐random sequences for numerical integration improves on the convergence rates of standard pseudorandom Monte Carlo integration and retains this performance in high dimensions.

Nevertheless, further development is needed. When producing estimates for a specific target population, we have assumed that the joint covariate distribution in the target population is known, and thus any estimation uncertainty for the covariate distribution is not accounted for. We have focused on a binary outcome with a probit link function, and application to continuous data and other link functions is natural. However, further research is required to extend ML‐NMR to other types of data such as multinomial or time‐to‐event data, where the correct form of the aggregate level likelihood is less obvious. MAIC methodology has already proven popular for survival analysis, and an extension of ML‐NMR to survival data would further increase the applicability of this new method. Simulation studies are necessary to assess the performance of ML‐NMR in a wide variety of scenarios and robustness to failure in assumptions, in comparison with other methods. Simulation studies may also compare estimates that are obtained by using ML‐NMR with a mixture of aggregate data and IPD studies with those from an IPD NMA with IPD for all studies. We have conducted a comprehensive simulation study, which shows that ML‐NMR does perform well under a wide range of scenarios, as long as there are no unobserved effect modifiers and the shared effect modifier assumption holds (if used).

Although motivated by a commonly occurring scenario in health technology appraisal, the proposed method to fit models combining individual and aggregate level data by using numerical integration is applicable beyond the context of NMA and indirect comparisons of randomized controlled trials. Notably, this approach is equally applicable to the ecological inference literature, where algebraic solutions to integrating an individual level model over a population have thus far been pursued (Jackson *et al*., 2006, 2008; Salway and Wakefield, 2005), and which inspired the first applications to NMA (Jansen, 2012). Moreover, the application of this approach to incorporate data from observational or single‐arm studies is a key area for further research, albeit relying on much stronger assumptions (Phillippo *et al*., 2016, 2018). The problem of synthesizing information that is reported at different levels of aggregation is common to many areas, and the methods that are developed in this paper are likely to be applicable to this general problem in a variety of contexts.

## Supporting information

‘Appendix’.
