# Supplementary material for: Multilevel network meta‐regression for population‐adjusted treatment comparisons
Source: J R Stat Soc Ser A Stat Soc. 2020 Jun 7;183(3):1189–210. doi: 10.1111/rssa.12579 (PMC7362893; doi:10.1111/rssa.12579)
Supplement: Supplementary file 1 — ‘Appendix’. [file RSSA-183-1189-s001.pdf]

A. Appendix

A.1. Analytic derivation of the aggregate-level model for probit regression

We derive the aggregate-level model for probit regression, where $g(\cdot)$ is the probit link function $g(p) = \Phi^{-1}(p)$ (the standard Normal inverse CDF). To begin with, let us consider the one-parameter Binomial approximation to the aggregate likelihood:

$$\begin{aligned} y_{\bullet jk} &\sim \mathrm{Bin}(N_{jk}, \bar{p}_{jk}), \\ \bar{p}_{jk} = \theta_{\bullet jk} &= \int_{\mathfrak{X}} \Phi\big(\eta_{jk}(\boldsymbol{x})\big) f_{jk}(\boldsymbol{x})\, d\boldsymbol{x}. \end{aligned} \tag{A.1}$$

This integral does not always have a closed form solution; however, if the covariates are multivariate-Normal, $\boldsymbol{x}_{jk} \sim \mathrm{MVN}(\boldsymbol{m}_{jk}, \Sigma_{jk})$, then we have

$$\bar{p}_{jk} = \Phi\left(\eta_{jk}(\boldsymbol{m}_{jk}) \left(1 + (\boldsymbol{\beta}_1 + \boldsymbol{\beta}_{2,k})^{\mathsf{T}} \Sigma_{jk} (\boldsymbol{\beta}_1 + \boldsymbol{\beta}_{2,k})\right)^{-\frac{1}{2}}\right). \tag{A.2}$$

The multivariate Normal scenario offers some insight into the relationship between ML-NMR and the naïve approach of "plugging in" mean covariate values. Equation (A.2) can be viewed as an adjustment to the naïve model, with the adjustment term involving the covariate covariance matrix Σ_{jk} accounting for the integration over the population. Examining the adjustment term, we notice that this depends upon

- The covariance of prognostic variables and effect modifiers, and the strength of each, through $\boldsymbol{\beta}_1^{\mathsf{T}} \Sigma_{jk} \boldsymbol{\beta}_{2,k}$. This is large when highly prognostic variables are correlated with strong effect modifiers, or when strong effect modifiers which are also highly prognostic take a wide range of values in the j-th study population (the population variance is large).

- The covariance of effect modifiers and their strength, through $\boldsymbol{\beta}_{2,k}^{\mathsf{T}} \Sigma_j \boldsymbol{\beta}_{2,k}$. This is large when strong effect modifiers are highly correlated, or when strong effect modifiers take on a wide range of values in the j-th study population (the population variance is large).

This lends theoretical justification to intuitive thinking for when effect modification may lead to aggregation bias. In particular, aggregation bias is expected to be small if either effect modifiers are weak, or the within-study variance of effect modifiers is small. The covariance inequality bounds the covariance between two variables by their standard deviations, $|\mathrm{cov}(X_1, X_2)| \leq \sqrt{\mathrm{var}(X_1)\,\mathrm{var}(X_2)}$; small within-study variance in the effect modifiers is therefore sufficient to also limit the aggregation bias due to correlations.

To use the two-parameter Binomial approximation to the Poisson Binomial likelihood (see equation 4), we additionally need to evaluate $\bar{p^2}_{jk}$. The derivation is similar to that for $\bar{p}_{jk}$, except now the integration is over the squared linear predictor:

$$\bar{p^2}_{jk} = \int_{\mathfrak{X}} g^{-1}\big(\eta_{jk}(\boldsymbol{x})\big)^2 f_{jk}(\boldsymbol{x})\, d\boldsymbol{x}. \tag{A.3}$$

For probit regression with $g^{-1}(\cdot) = \Phi(\cdot)$, and multivariate Normal covariates, the integration

is carried out as follows:

$$\begin{aligned}\bar{p^2}_{jk} &= \int_{\mathfrak{X}} \Phi(\eta_{jk}(\boldsymbol{x}))^2 f_{jk}(\boldsymbol{x})\,d\boldsymbol{x} \\ &= \Phi\left(\eta_{jk}(\boldsymbol{m}_{jk})\left(1 + (\boldsymbol{\beta}_1 + \boldsymbol{\beta}_{2,k})^{\mathsf{T}} \Sigma_{jk} (\boldsymbol{\beta}_1 + \boldsymbol{\beta}_{2,k})\right)^{-\frac{1}{2}}\right) \\ &\quad - 2\,\mathrm{T}\left(\eta_{jk}(\boldsymbol{m}_{jk})\left(1 + (\boldsymbol{\beta}_1 + \boldsymbol{\beta}_{2,k})^{\mathsf{T}} \Sigma_{jk} (\boldsymbol{\beta}_1 + \boldsymbol{\beta}_{2,k})\right)^{-\frac{1}{2}},\right. \\ &\qquad\qquad \left.\left(1 + 2(\boldsymbol{\beta}_1 + \boldsymbol{\beta}_{2,k})^{\mathsf{T}} \Sigma_{jk} (\boldsymbol{\beta}_1 + \boldsymbol{\beta}_{2,k})\right)^{-\frac{1}{2}}\right)\end{aligned} \tag{A.4}$$

where $\mathrm{T}(\cdot,\cdot)$ is Owen's T function, which may be evaluated using fast numerical methods (e.g. quadrature).

For skew covariates, evaluation of the aggregation integral (1d) is troublesome: analytic integration for both log-Normal and Gamma distributed covariates is not straightforward. In an ecological context, Salway and Wakefield (2005) noted that estimates remained biased when skew covariates were treated as Normal (although less so than the naïve approach), and uncertainty was underestimated.

A.2. Analytic integration with the logit link function

When using the logit link function $g(p) = \log\left(\frac{p}{1-p}\right)$, as in logistic regression, the integral (1d) does not have a closed form solution. Previous authors have suggested approximating the logit link by a probit (Salway and Wakefield, 2005; Demidenko, 2004), for example

$$\text{One-probit approx.:}\quad \operatorname{logit}^{-1}(\eta) \approx \Phi\left(\tfrac{16\sqrt{3}}{15\pi}\eta\right) \tag{A.5a}$$

$$\text{Two-probit approx.:}\quad \operatorname{logit}^{-1}(\eta) \approx 0.4353\,\Phi\left(\frac{\eta}{2.2967}\right) + 0.5647\,\Phi\left(\frac{\eta}{1.3017}\right) \tag{A.5b}$$

The two-probit approximation is particularly robust, and still results in a proper likelihood distribution as the coefficients sum to one. Higher order approximations are possible and can be even more accurate, but do not necessarily result in proper likelihood distributions.

Continuing with the one-probit approximation first, the average success probability on treatment k in the j-th study is then approximately

$$\bar{p}_{jk} \approx \int_{\mathfrak{X}} \Phi\left(\tfrac{16\sqrt{3}}{15\pi}\eta_{jk}(\boldsymbol{x})\right) f_{jk}(\boldsymbol{x})\,d\boldsymbol{x}. \tag{A.6}$$

If the covariates are multivariate-Normal, $\boldsymbol{x}_j \sim \mathrm{MVN}(\boldsymbol{m}_j, \Sigma_j)$, then we have (Salway and Wakefield, 2005)

$$\bar{p}_{jk} \approx \operatorname{logit}^{-1}\left(\eta_{jk}(\boldsymbol{m}_j)\left(1 + \left(\tfrac{16\sqrt{3}}{15\pi}\right)^2 (\boldsymbol{\beta}_1 + \boldsymbol{\beta}_{2,k})^{\mathsf{T}} \Sigma_j (\boldsymbol{\beta}_1 + \boldsymbol{\beta}_{2,k})\right)^{-\frac{1}{2}}\right). \tag{A.7}$$

Derivation proceeds similarly for the two-probit approximation with multivariate-Normal covariates $\boldsymbol{x}_j$,

$$\bar{p}_{jk} \approx 0.4353\,\Phi\left(\frac{\eta_{jk}(\boldsymbol{m}_j)}{2.2967}\left(1 + 2.2967^{-2}(\boldsymbol{\beta}_1 + \boldsymbol{\beta}_{2,k})^{\mathsf{T}}\Sigma_j(\boldsymbol{\beta}_1 + \boldsymbol{\beta}_{2,k})\right)^{-\frac{1}{2}}\right) + 0.5647\,\Phi\left(\frac{\eta_{jk}(\boldsymbol{m}_j)}{1.3017}\left(1 + 1.3017^{-2}(\boldsymbol{\beta}_1 + \boldsymbol{\beta}_{2,k})^{\mathsf{T}}\Sigma_j(\boldsymbol{\beta}_1 + \boldsymbol{\beta}_{2,k})\right)^{-\frac{1}{2}}\right) \quad \text{(A.8)}$$

A.3. *Imputing a correlation structure with copulæ*

We typically only have access to published marginal covariate information for the AgD trials, and therefore have no information on the correlation structure between the covariates. Rather than assuming that all correlations between covariates in the AgD are zero (which may be unreasonable), we can instead assume that the correlation structures are similar in AgD and IPD. To account for this, we compute a correlation matrix Ω for the AgD trials from the IPD trials (or a representative subset), and impose this upon the Sobol' integration points $\tilde{\boldsymbol{u}}$ using a Gaussian copula (Nelsen, 2006). This is equivalent to applying the inverse CDF $\Phi_\Omega^{-1}(\cdot)$ of the multivariate Normal with correlation matrix Ω, and then the standard multivariate Normal CDF $\Phi(\cdot)$,

$$\tilde{\boldsymbol{u}}^* = \Phi(\Phi_\Omega^{-1}(\tilde{\boldsymbol{u}})),$$

to obtain correlated Sobol' points $\tilde{\boldsymbol{u}}^*$. (In practice this is computed component-wise conditionally; we use the implementation in the R package `copula` (Yan, 2007).) The correlated Sobol' points are then transformed using the inverse CDF method to match the marginal covariate distributions reported in the AgD trials,

$$\tilde{x}_{jk;l} = F_{jk;l}^{-1}(\tilde{u}_l^*) \quad \text{for } l = 1, \dots, L,$$

where $F_{jk;l}^{-1}(\cdot)$ is the inverse CDF of the marginal distribution assumed for the l-th covariate $\tilde{x}_{jk;l}$ on treatment k in study j. The resulting integration points $\tilde{\boldsymbol{x}}_{jk}$ capture the correlations between the covariates (e.g. longer duration of psoriasis is correlated with having previous systemic treatment) whilst preserving the marginal distribution for each covariate.

A.4. *Assessing inconsistency*

ML-NMR, like standard IPD and AgD NMA, makes an assumption of consistency that is enforced through a set of consistency equations $d_{ab} = d_b - d_a$. For ML-NMR, consistency applies to both the individual-level treatment effects, $\gamma_{ab} = \gamma_b - \gamma_a$, and the effect modifier interactions, $\boldsymbol{\beta}_{2,ab} = \boldsymbol{\beta}_{2,b} - \boldsymbol{\beta}_{2,a}$. The causes of inconsistency in ML-NMR are the same as the causes of heterogeneity described in section 2.4. For example, there may be effect modifiers that have not been included in the model or other model misspecification, the assumed joint covariate distributions used to adjust the results from aggregate studies may be incorrect, or the shared effect modifier assumption (if it was used) may be invalid. Attempts may be made to rectify these issues in a revised model—if data permits—and the revised model may then be assessed for inconsistency. To assess inconsistency, we can use the same approaches as standard IPD and AgD NMA—such as the unrelated mean effects model (Dias et al., 2011b), and node-splitting models (Dias et al., 2010).

Unrelated mean effects

The unrelated mean effects (UME) model (Dias et al., 2011b) treats all contrasts—both basic (i.e. against treatment 1) and functional—as independent parameters, without imposing consistency. The UME model needs to be written with the study-specific baselines referring to a reference *arm* in each trial (the "baseline shift" parameterisation), rather than the reference treatment 1, since the latter imposes consistency implicitly. For random effects ML-NMR (9), we must also consider the EM interaction terms, and whether or not we allow these to be inconsistent too (Donegan et al., 2017). The linear predictor and random effects structure of the UME model for RE ML-NMR are written as

$$\eta_{jk}(\boldsymbol{x}) = \mu_j^{(t_1)} + \boldsymbol{x}^{\top}(\boldsymbol{\beta}_1 + \boldsymbol{\beta}_{2,t_1 k}) + \delta_{jt_1 k} \tag{A.9a}$$

$$\delta_{jt_1 k} \sim \mathrm{N}(\gamma_{t_1 k}, \tau^2) \tag{A.9b}$$

$$\mathrm{cor}(\delta_{jt_1 a}, \delta_{jt_1 b}) = 0.5 \tag{A.9c}$$

for a study j with treatment t_1 in arm 1, where $\mu_j^{(t_1)}$ is the study-specific baseline with respect to t_1.

If we apply the consistency equations to the EM interactions, $\boldsymbol{\beta}_{2,ab} = \boldsymbol{\beta}_{2,b} - \boldsymbol{\beta}_{2,a}$, then (A.9) only relaxes consistency in the treatment effects. There can also be inconsistency in the EM interactions (Donegan et al., 2017); to assess this as well, we instead place independent prior distributions on $\boldsymbol{\beta}_{2,ab}$, such as $\mathrm{N}(0, \sigma^2_{\beta_2})$ for a suitable prior variance $\sigma^2_{\beta_2}$. However, this requires sufficient data on each contrast to estimate independent interactions. This may not be possible, for example if there are contrasts which are only informed by a small number of AgD studies.

An intermediate approach is possible when the shared EM assumption (section 2.3) is used to fit the ML-NMR model, so that the regression coefficients $\boldsymbol{\beta}_{2,k}$ for a set of treatments $k \in \mathcal{T}$ are all equal. In this case, we can use the shared EM assumption—which implies that certain interactions are zero or equal to each other—and allow the remaining interactions to be inconsistent. To achieve this, consider (without loss of generality) that every treatment is assigned to a mutually exclusive set $\mathcal{T}_1, \mathcal{T}_2, \dots$ (some treatments may be in a set by themselves). Then, using the shared EM assumption, EM interactions for contrasts between any two treatments within a given set $\mathcal{T}$ are equal to zero, $\boldsymbol{\beta}_{2,ab} = \boldsymbol{0}$ for any two treatments $a, b \in \mathcal{T}$. EM interactions for contrasts between treatments in any two different sets $\mathcal{T}_1, \mathcal{T}_2$ are equal, $\boldsymbol{\beta}_{2,a_1 a_2} = \boldsymbol{\beta}_{2,b_1 b_2}$ for any treatments $a_1, b_1 \in \mathcal{T}_1$ and $a_2, b_2 \in \mathcal{T}_2$, and are assigned a prior distribution such as $\mathrm{N}(0, \sigma^2_{\beta_2})$. This allows us to assess inconsistency of the shared EM interactions, and such a model should always be identifiable when the corresponding standard (consistency) ML-NMR model with shared EM interactions is identifiable.

In any case, evidence for inconsistency is then based on comparing the model fit (e.g. using residual deviance and DIC) between the ML-NMR model assuming consistency and the UME model without consistency.

Node-splitting

The node-splitting approach for network meta-regression models (Donegan et al., 2017) is naturally applicable to ML-NMR in the same manner as IPD network meta-regression. For a given contrast b' vs. a', the node-splitting model splits the estimation of the relative effect $\gamma_{a'b'}$ and effect modifier interactions $\boldsymbol{\beta}_{2,a'b'}$ into parameters estimated by direct evidence only, $\gamma^{\mathrm{D}}_{a'b'}$

and $\boldsymbol{\beta}^{\mathrm{D}}_{2,a'b'}$, and parameters estimated by the indirect evidence from the rest of the network, $\gamma^{\mathrm{I}}_{a'b'}$ and $\boldsymbol{\beta}^{\mathrm{I}}_{2,a'b'}$. To achieve this, the random effects ML-NMR model (9) remains the same for studies not including both a' and b' treatment arms. For studies including both a' and b' treatment arms, we choose to re-parameterise the model with a' as the reference treatment within these studies. Mathematically, we write out the linear predictor and random effects for this node-splitting model as

For studies without both a' and b' treatment arms:

$$\eta_{jk}(\boldsymbol{x}) = \mu_j^{(1)} + \boldsymbol{x}^{\top}(\boldsymbol{\beta}_1 + \boldsymbol{\beta}_{2,k}) + \delta_{jk} \tag{A.10a}$$

$$\delta_{jk} \sim \mathrm{N}(\gamma_k, \tau^2) \tag{A.10b}$$

$$\mathrm{cor}(\delta_{ja}, \delta_{jb}) = 0.5 \tag{A.10c}$$

For studies with both a' and b' treatment arms:

$$\eta_{ja'}(\boldsymbol{x}) = \mu_j^{(a')} + \boldsymbol{x}^{\top}\boldsymbol{\beta}_1 \tag{A.10d}$$

$$\eta_{jb'}(\boldsymbol{x}) = \mu_j^{(a')} + \boldsymbol{x}^{\top}(\boldsymbol{\beta}_1 + \boldsymbol{\beta}^{\mathrm{D}}_{2,a'b'}) + \delta_{ja'b'} \tag{A.10e}$$

$$\eta_{jk}(\boldsymbol{x}) = \mu_j^{(a')} + \boldsymbol{x}^{\top}(\boldsymbol{\beta}_1 + \boldsymbol{\beta}_{2,k} - \boldsymbol{\beta}_{2,a'}) + \delta_{ja'k} \quad \text{for } k \neq a', b' \tag{A.10f}$$

$$\delta_{ja'b'} \sim \mathrm{N}(\gamma^{\mathrm{D}}_{a'b'}, \tau^2) \tag{A.10g}$$

$$\delta_{ja'k} \sim \mathrm{N}(\gamma_k - \gamma_{a'}, \tau^2) \quad \text{for } k \neq a', b' \tag{A.10h}$$

$$\mathrm{cor}(\delta_{ja'a}, \delta_{ja'b}) = 0.5 \quad \text{for } a, b \neq a', b' \tag{A.10i}$$

$$\mathrm{cor}(\delta_{ja'k}, \delta_{ja'b'}) = 0 \quad \text{for } k \neq a', b' \tag{A.10j}$$

where the re-parameterised study-specific baselines with respect to treatment a' are denoted by $\mu_j^{(a')}$, and we write the study-specific baselines with respect to treatment 1 as $\mu_j^{(1)} = \mu_j$ for additional clarity. As usual we set $\gamma_1 = \delta_{j1} = 0$ and $\boldsymbol{\beta}_{2,1} = \mathbf{0}$. If there are multi-arm studies with both a' and b' treatment arms, then the other random effects $\delta_{ja'k}$ with $k \neq b'$ are uncorrelated with $\delta_{ja'b'}$, but are still correlated between themselves with $\mathrm{cor}(\delta_{ja'a}, \delta_{ja'b}) = 0.5$ for $a, b \neq a', b'$ (assuming homogeneous τ^2). The indirect estimates $\gamma^{\mathrm{I}}_{a'b'}$ and $\boldsymbol{\beta}^{\mathrm{I}}_{2,a'b'}$ are obtained from the consistency equations

$$\begin{aligned} \gamma^{\mathrm{I}}_{a'b'} &= \gamma_{b'} - \gamma_{a'}, \\ \boldsymbol{\beta}^{\mathrm{I}}_{2,a'b'} &= \boldsymbol{\beta}_{2,b'} - \boldsymbol{\beta}_{2,a'}. \end{aligned} \tag{A.11}$$

The node-splitting model as written in (A.10) splits the EM interaction terms for all covariates at once. Alternatively, a separate node-splitting model could be fitted for each covariate in turn, where $\boldsymbol{\beta}^{\mathrm{D}}_{2,a'b'}$ is broken down into a split interaction for one covariate, $\beta^{\mathrm{D}}_{2,a'b';l}$, and the consistency equations are applied for the remaining covariates $\beta_{2,a'b';l} = \beta_{2,b';l} - \beta_{2,a';l}$. The latter approach may be more tractable in scenarios with smaller amounts of data on the b' vs. a' contrast and/or large numbers of effect modifying covariates, since there are only 2 more parameters than the standard RE ML-NMR model ($\gamma^{\mathrm{D}}_{a'b'}$ and $\beta^{\mathrm{D}}_{2,a'b';l}$), as opposed to $L + 1$ more when splitting all EM interactions at once ($\gamma^{\mathrm{D}}_{a'b'}$ and $\boldsymbol{\beta}^{\mathrm{D}}_{2,a'b'}$), where L is the number of covariates.

Furthermore, there may be insufficient data on the b' vs. a' contrast even to node-split the EM interaction terms one covariate at a time, for example if the direct evidence consists of only

a small number of AgD studies. In this case, we may be able to assess inconsistency in the treatment contrast $\gamma_{a'b'}$ by node-splitting into $\gamma^{\mathrm{D}}_{a'b'}$ and $\gamma^{\mathrm{I}}_{a'b'}$, but not in the EM interactions, keeping the consistency equations $\boldsymbol{\beta}_{2,a'b'} = \boldsymbol{\beta}_{2,b'} - \boldsymbol{\beta}_{2,a'}$.

As with the unrelated mean effects model (appendix A.4), evidence for inconsistency based on comparing the model fit (e.g. using residual deviance and DIC) between the ML-NMR model with and without node-splitting, and also checking whether the heterogeneity variance τ^2 is reduced. The graphical summaries described by Donegan et al. (2017) may also be utilised.

A.5. Results for plaque psoriasis example

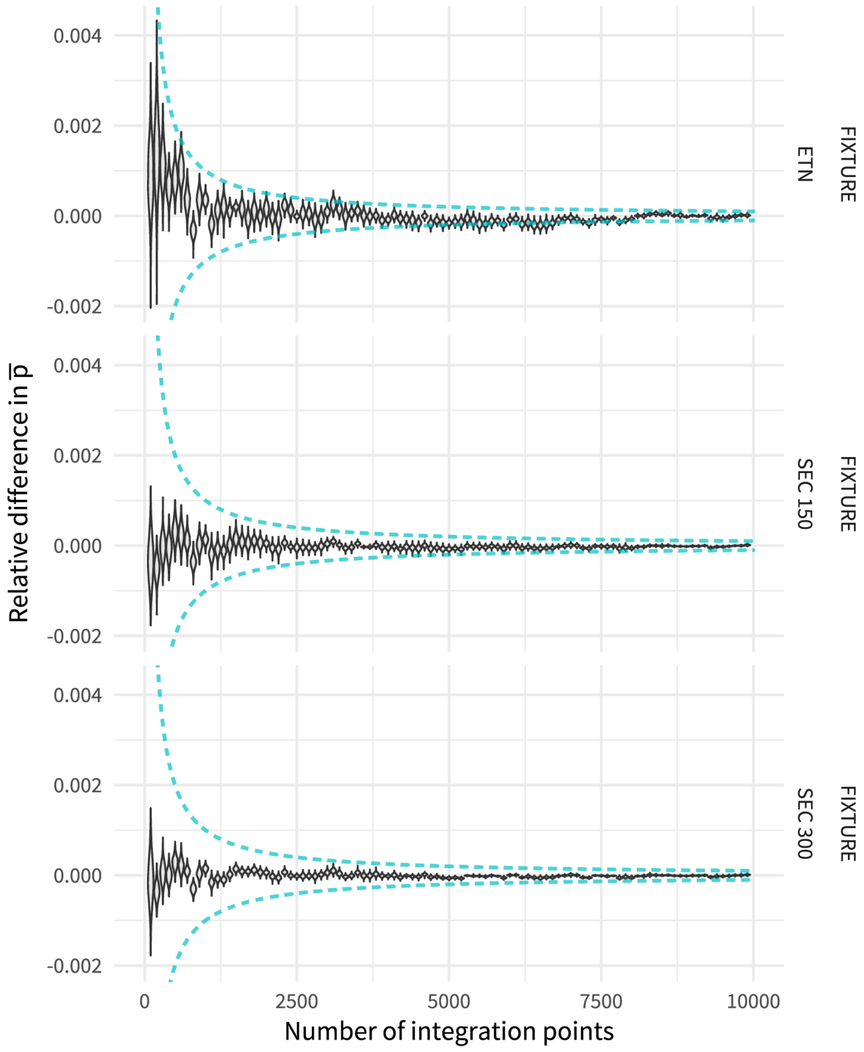

Fig. A.1. Empirical integration error for $\bar{p}$ over the entire posterior of the model parameters, estimated as a relative difference from the final estimate with 10,000 integration points (at each posterior sample). The dashed line is $\pm\tilde{N}^{-1}$, showing that the integration error rate is of this order.

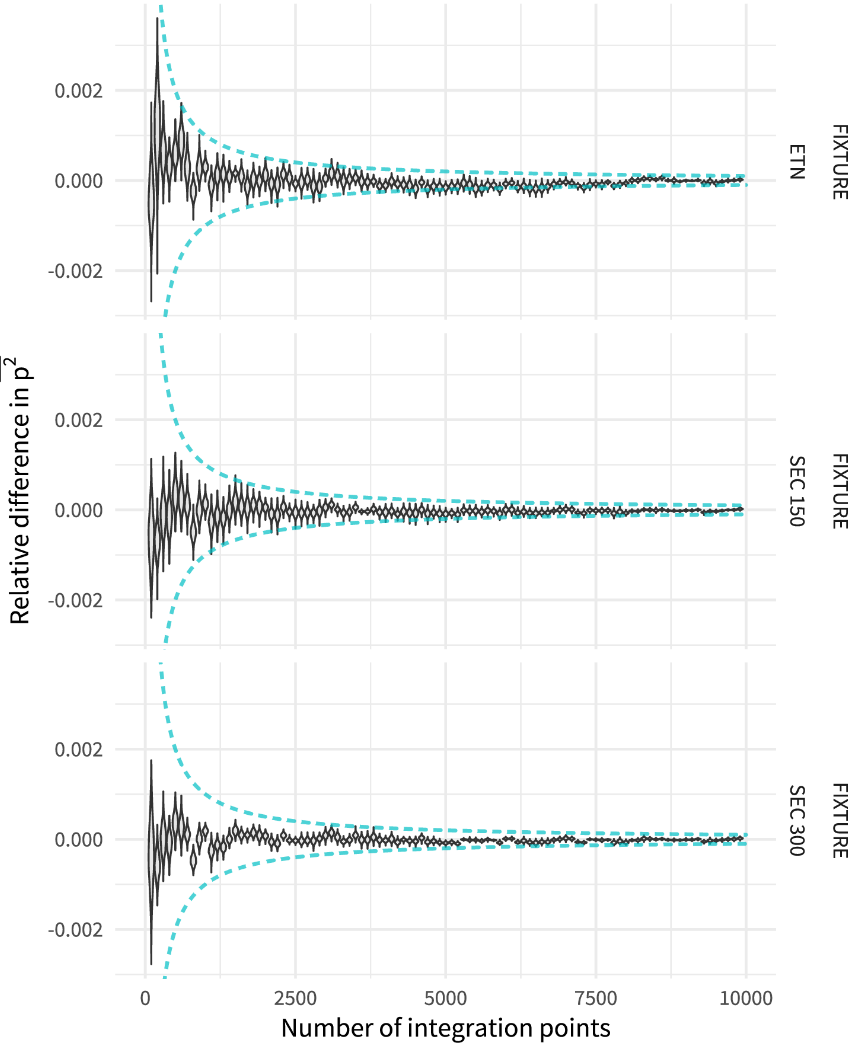

Fig. A.2. Empirical integration error for $\bar{p^2}$ over the entire posterior of the model parameters, estimated as a relative difference from the final estimate with 10,000 integration points (at each posterior sample). The dashed line is $\pm\tilde{N}^{-1}$, showing that the integration error rate is of this order.

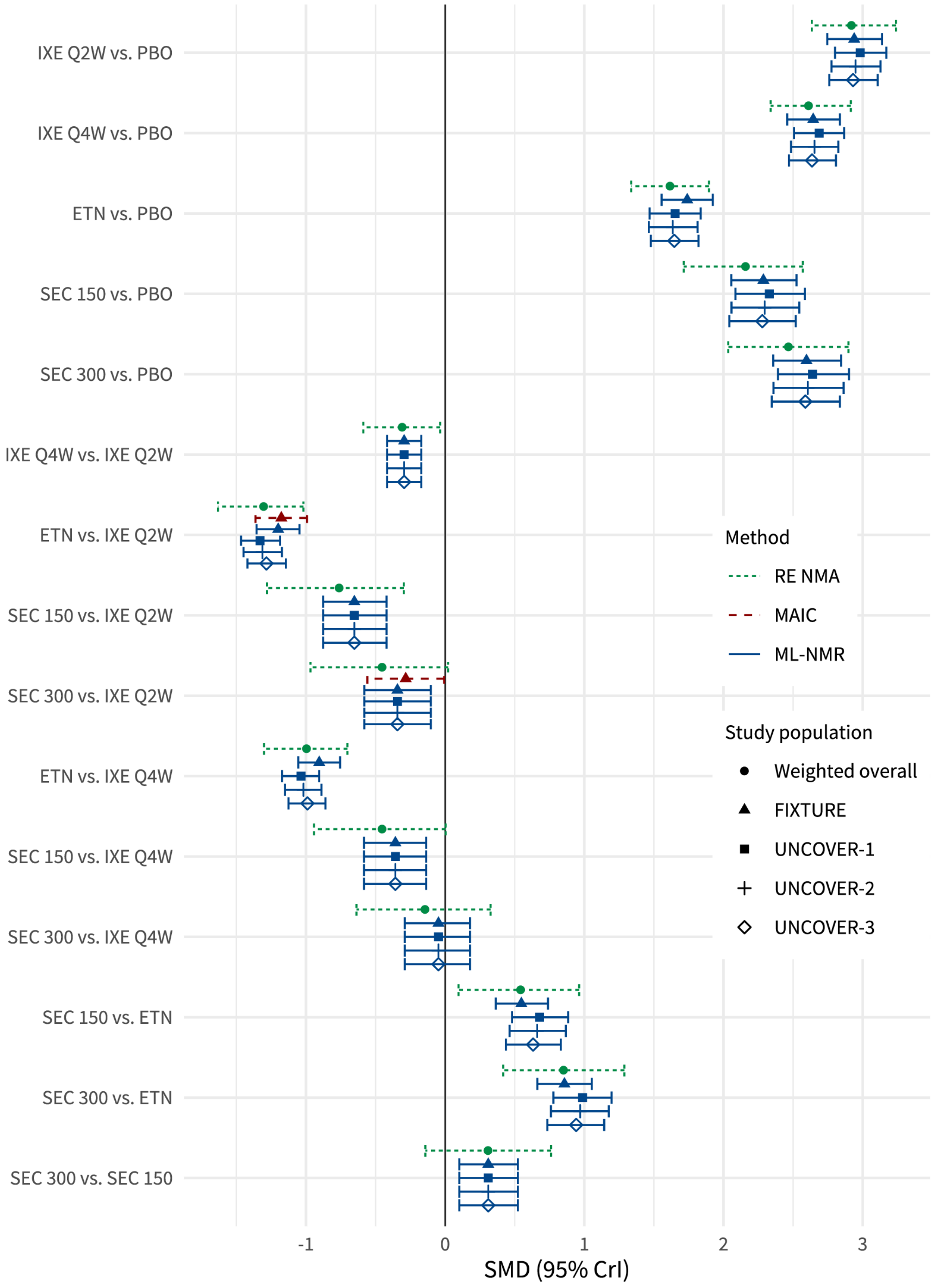

Fig. A.3. Estimated contrasts at the population level, for each pair of treatments in each study population. Note that the interval for MAIC is a 95% Confidence Interval, as MAIC is a frequentist method.

Table A.1. Estimated proportion of individuals achieving PASI 75 on each treatment in each study population, along with 95% Credible Intervals, for each method. The observed proportions are accompanied by 95% Confidence Intervals, calculated on the probit scale.
* MAIC estimate is 84.74 (78.54, 89.62).

		Treatment					
Study population	Method	Placebo	Ixekizumab Q2W	Ixekizumab Q4W	Etanercept	Secukinumab 150 mg	Secukinumab 300 mg
FIXTURE	Observed	4.94 (2.85, 7.90)	-	-	43.96 (38.47, 49.56)	66.97 (61.59, 72.05)	77.09 (72.11, 81.56)
	ML-NMR	3.83 (2.58, 5.38)	85.93* (81.58, 89.64)	78.46 (72.84, 83.27)	46.10 (40.93, 51.11)	66.96 (61.82, 71.72)	77.07 (72.72, 81.34)
UNCOVER-1	Observed	3.94 (2.31, 6.24)	89.15 (85.83, 91.91)	82.64 (78.73, 86.09)	-	-	-
	ML-NMR	4.76 (3.37, 6.47)	88.72 (86.48, 90.79)	82.19 (79.10, 85.11)	46.57 (40.92, 52.22)	71.59 (63.69, 78.78)	80.79 (73.72, 86.93)
UNCOVER-2	Observed	2.41 (0.66, 6.05)	90.00 (86.37, 92.94)	77.46 (72.68, 81.75)	41.74 (36.57, 47.04)	-	-
	ML-NMR	4.06 (2.80, 5.66)	86.59 (84.20, 88.88)	79.31 (76.35, 82.11)	43.05 (39.23, 46.97)	67.92 (60.01, 75.19)	77.83 (70.69, 84.00)
UNCOVER-3	Observed	7.29 (4.04, 11.93)	87.50 (83.77, 90.64)	85.30 (81.34, 88.70)	53.40 (48.26, 58.49)	-	-
	ML-NMR	6.23 (4.48, 8.35)	90.33 (88.35, 92.20)	84.41 (81.74, 86.94)	51.69 (47.55, 55.94)	74.52 (67.41, 80.93)	83.13 (76.49, 88.31)
